# Application of Advanced Imaging to Prostate Cancer Diagnosis and Management: A Narrative Review of Current Practice and Unanswered Questions

**DOI:** 10.3390/jcm13020446

**Published:** 2024-01-13

**Authors:** Elizabeth L. McKone, Elsa A. Sutton, Geoffrey B. Johnson, Ryan M. Phillips

**Affiliations:** 1 Department of Radiation Oncology, Mayo Clinic, Rochester, MN 55905, USA; 2 Department of Radiology, Nuclear Medicine Division, Mayo Clinic, Rochester, MN 55905, USA

**Keywords:** prostate cancer, prostate imaging, MRI, molecular Imaging, positron emission tomography (PET)

## Abstract

Major advances in prostate cancer diagnosis, staging, and management have occurred over the past decade, largely due to our improved understanding of the technical aspects and clinical applications of advanced imaging, specifically magnetic resonance imaging (MRI) and prostate-cancer-specific positron emission tomography (PET). Herein, we review the established utility of these important and exciting technologies, as well as areas of controversy and uncertainty that remain important areas for future study. There is strong evidence supporting the utility of MRI in guiding initial biopsy and assessing local disease. There is debate, however, regarding how to best use the imaging modality in risk stratification, treatment planning, and assessment of biochemical failure. Prostate-cancer-specific PET is a relatively new technology that provides great value to the evaluation of newly diagnosed, treated, and recurrent prostate cancer. However, its ideal use in treatment decision making, staging, recurrence detection, and surveillance necessitates further research. Continued study of both imaging modalities will allow for an improved understanding of their best utilization in improving cancer care.

## 1. Introduction

Despite rapid and significant advances in our understanding of all aspects of prostate cancer, it remains the most diagnosed malignancy among US men, accounting for approximately 290,000 new diagnoses and 35,000 deaths [1]. Prostate cancer is far from a uniform diagnosis. Itcan be sufficiently indolent to warrant surveillance alone, or it can be so aggressive and disseminated at diagnosis that aggressive multi-disciplinary cancer care is critical to maximizing outcomes. No advances have furthered our contemporary understanding and navigation of this continuum more significantly than the development of advanced imaging techniques using magnetic resonance imaging (MRI) and positron emission tomography (PET); both technologies provide access to unique clinical data for diagnosis, treatment, and monitoring as summarized in Table 1, but routine utilization is variable across practices and consensus recommendations for best practices are far from universally agreed upon.

## 2. The Role of MRI in Contemporary Prostate Cancer Management

### 2.1. MRI for Prostate Cancer Diagnosis, Staging, and Risk Stratification

Current guidelines vary regarding the routine use of pre-biopsy MRI in the initial evaluation of suspected prostate cancer. Discussion of MRI as a component of initial workup is limited and vague in the NCCN guidelines [2] and the American Urological Association/American Society for Radiation Oncology guidelines [3] state that there are insufficient data to recommend routine MRI in every biopsy-naïve patient. Conversely, updated guidelines from the European Association of Urology [4] and UK National Institute of Health [5] do support MRI in initial workup. 

Much of the utility of MR prior to biopsy lies in its ability to highlight areas of suspected disease in the prostate, providing a target for biopsy. The benefits of MR-guided biopsy in comparison to standard biopsy were demonstrated by the PRECISION trial, a multicenter randomized trial comparing the diagnosis of clinically significant prostate cancer among men who received standard TRUS-guided biopsy with men who underwent MRI-targeted biopsy [6]. They found that MRI-guided biopsy was able to detect clinically significant cancer more frequently than TRUS-guided biopsy (38% vs. 26% of cases, *p* = 0.005). Similarly, a 2015 prospective cohort study of 1003 men undergoing both standard sextant biopsy and MR-targeted biopsy found MR-targeted biopsy to diagnose 30% more high-risk cancers (*p* < 0.001) [7]. These findings, however, do not negate the need for sextant biopsy. The MRI first trial prospectively evaluated biopsy specimens taken from men who underwent both TRUS- and MR-guided biopsies [8]. They found that 5.2% of clinically significant cancers would be missed with MR-guided biopsy alone, and 7.6% of cancers would be missed with TRUS-guided biopsy alone. Of note, while the NCCN guidelines do not expressly recommend MR-guided biopsy, they do clarify how to incorporate biopsy cores obtained by MR-guided biopsy in risk group stratification. Guidelines state that an MRI-targeted lesion found to be positive should be considered as a single positive core, regardless of the number or percentage of cores positive.

MRI is also useful in identifying the local extent of disease. Traditionally, digital rectal examination (DRE) was the method of choice for local assessment of prostate cancer and remains the sole technique permitted by the AJCC 8th edition for T-staging, with the manual specifically mentioning that imaging information should not be used [9]. However, recent large clinical trials such as STAMPEDE and NRG GU009 have allowed the inclusion of MRI for T-staging, reflecting a shift in clinical practice from physical exam-based to image-based staging [10] and raising the question: how does MRI perform in comparison to DRE?

There are several studies comparing the diagnostic accuracy of MRI to DRE, specifically in their abilities to detect extraprostatic extension. One such multi-institutional Dutch study analyzed the pre-surgical MRIs and DREs of 1683 men who later underwent prostatectomy [11]. Using the prostatectomy pathology as the gold standard, MRI was found to have significantly higher sensitivity for detecting T3a or higher disease than DRE (51% vs. 12%, *p* < 0.001). Although MRI did have fairly good specificity (82%) for T3a or higher disease, it was not as specific as DRE (97%). Another retrospective analysis of men who underwent both DRE and MRI prior to prostatectomy had similar findings, with MRI having higher sensitivity than DRE (59% vs. 41%, *p* < 0.01) in the detection of extraprostatic disease, but lower specificity (69% vs. 95%, *p* < 0.01) [12]. Of note, the interobserver variability of DRE in detecting suspicious lesions is greater than that of MRI (*k* = 0.22 vs. *k* = 0.57) [13,14]. 

The high sensitivity but relatively low specificity of MR for extraprostatic extension calls into question whether basing treatment decisions on MR findings contributes to overtreatment. The aforementioned Dutch study found that incorporating MR findings into staging downstaged only 3% of patients but upstaged 29% (largely due to extraprostatic extension detected on MR). When stratifying by MRI-based risk grouping, there was less difference in biochemical recurrence-free survival (BRFS) among risk groups than when stratifying by DRE-based risk grouping. This may indicate that extracapsular extension that can only be detected on MRI is not of prognostic importance. Alternatively, however, the smaller difference in BRFS among MR-based risk groups may be attributed to an appropriate escalation of care that would not have otherwise been carried out without the additional information provided by MR. 

More detailed information on extraprostatic extension not readily assessed by DRE may also help inform treatment decisions. For example, a large retrospective database analysis found that men who had focal extraprostatic extension on MRI had better BRFS than men who had extensive extraprostatic extension [15]. Another retrospective study found that the radial distance of prostate cancer from the capsule predicted PSA recurrence [16]. Thus, we can garner more nuanced prognostic information with MRI than the binary presence or absence of extraprostatic extension assessed on DRE. Such information may also be used to guide decision-making regarding the appropriateness of focal therapies such as high-intensity focused ultrasound or cyroablation [17]. These approaches present a promising alternative to radical prostatectomy or definitive radiotherapy, but expectations of efficacy and toxicity—and by extention appropriateness of focal therapy—may vary based on the extent of intraprostatic disease. Although it is unclear if the additional information detected only on MRI warrants treatment escalation, MR can provide detailed insight into the extent of a patient’s disease that may contribute to treatment decision making, along with other factors such as PSA, Gleason score, emerging technologies such as genomic classifiers and digital pathology, and the patient’s desire for aggressive versus conservative treatment.

### 2.2. Use of MRI Prior to and during Active Surveillance

MRI can also be particularly useful in selecting patients who are good candidates for active surveillance (AS). An NCI study of 85 men who initially qualified for active surveillance reclassified 29% of men as not meeting the criteria based on confirmatory biopsy [18]. The study found that MR features including the number of lesions, lesion density, and lesion suspicion were significantly associated with confirmatory biopsy disqualification from AS. Furthermore, a French bi-institutional study found that 10% of patients originally considered for active surveillance were disqualified due to findings on MR-fusion biopsy including Gleason 8 disease and/or cancer length within the biopsy core [19]. 

MRI also serves a role in monitoring patients on active surveillance. A cohort study of men on active surveillance who underwent MRIs at a median interval of every 2 years, as well as serial biopsies, found that a PIRADS score of 4–5 had 80% sensitivity, 62% specificity, 34% positive predictive value, and 93% negative predictive value for the Gleason upgrade [20]. Similarly, a review of men on active surveillance who underwent both MRI and repeat biopsies at follow-up visits found MR to have a 53% positive predictive value and 80% negative predictive value for Gleason progression [21]. The high negative predictive value of MRI can offer reassurance and reduce excessive biopsying, thus limiting the morbidity of active surveillance. However, this is not to say random biopsies should be omitted. A cohort study of over 200 men on active surveillance undergoing MRIs every 18 months and biopsies every 3 years found that, while biopsying only when progression is identified on MRI would avoid 681 biopsies per 1000 men, it would also miss Gleason grade 2+ disease in 169 of 1000 patients [22]. Although the emerging field of radiomics holds promise in improving the detection of clinically significant prostate cancer that would otherwise be missed by MRI, this approach is not yet broadly applicable to clinical practice [23]. Therefore, especially in fit men for who would desire aggressive treatment if indicated, random biopsies should be pursued regularly regardless of MRI findings.

Much like in the initial diagnosis of prostate cancer, MR-fusion biopsies of suspicious lesions on active surveillance improve the detection of clinically significant cancer. For example, the aforementioned study [21] found that the number needed to biopsy to detect Gleason progression was 8.74 for standard biopsy vs. 2.9 for MR-fusion biopsy, highlighting the high diagnostic yield of MRI in this setting. Another study found that higher grade cancer was detected in 11% of men on active surveillance who did not have an MRI-targetable lesion, whereas higher grade cancer was detected in 47% of men who underwent biopsy of a region of interest on MRI [24]. The NCCN guidelines concur with these findings, stating that MR-fusion biopsy improves the detection of high-grade cancers in men with suspicious lesions on MRI. Thus, MRI should be considered a standard component of monitoring on active surveillance. Of note, the frequency of MRI on active surveillance remains in question. While the NCCN guidelines recommend MRI be repeated up to yearly, the AUA states further study is necessary to determine the optimal timing of MRI.

### 2.3. Assessment of Recurrent Prostate Cancer by MRI

MRI is a helpful tool in assessing for local recurrence in the setting of biochemical failure, with use in both the post-prostatectomy and post-radiation settings. Although evidence regarding the ideal use of MRI in this space is limited, there are data demonstrating the value of MRI. For example, in a study of men with biochemical failure after prostatectomy, dynamic contrast-enhanced MRI was found to have 88% sensitivity and 100% specificity for local recurrence [25]. However, these results used positive TRUS-guided biopsy or response to radiation to define recurrence, not biochemical failure. Nearly half of the patients who had biochemical failure had no other signs of disease, which leaves in question how to evaluate and manage patients who have an undetectable source of PSA. 

The PSA level can influence the likelihood of a positive MRI in biochemical recurrence. The aforementioned study with an approximately 50% recurrence diagnosis rate included men with a mean PSA of 1.9. A review of over 700 men with biochemical failure (PSA ≥ 0.2) found that only 7% of men had positive MR [26]. However, increasingly elevated PSA levels led to an increasing diagnostic yield, with 10% of patients with PSA ≥ 0.4 demonstrating positive MRI, 13% of patients with PSA > 1, and 50% of patients with PSA > 10. In fact, the mean PSA in the group of patients with positive MRI was significantly higher than in patients with negative MRI (4.5 vs. 1.0, *p* < 0.01). Considering that the PSA level has been shown to correlate with the volume of disease, it is reasonable to infer that a minimum threshold of disease volume is necessary for the detection of recurrence on MRI [27]. Thus, if recurrent disease is not initially detectable on MRI, repeat imaging when PSA increases further may be useful. The timing of when to carry out subsequent MRI remains unclear.

MRI may also be used to detect local recurrence after primary treatment with radiation. After radiation, local recurrence can appear on T2W MRI as a hypointense nodular lesion and MRI has been shown to have modest accuracy in detecting recurrence. One study of men being evaluated for prostate cancer recurrence after radiation reports 63% accuracy for one radiologist and 71% for another when comparing MR interpretation to biopsy [28]. Another study of men who underwent salvage prostatectomy found the AUC of MRI tumor detection to be 0.75 in one reader and 0.61 in another using surgical pathology as the gold standard [29]. However, the optimal timing of MRI acquisition after biochemical failure in this setting is currently unclear.

In summary, MRI has many uses in the evaluation of prostate cancer, but guidelines have not yet reached consensus on how to incorporate MR information into clinical practice. MR-guided biopsy is a key adjunct to random biopsy that improves the detection of clinically significant prostate cancer. The high sensitivity afforded by MRI in the detection of extraprostatic extension contributes to upstaging, and controversy exists over whether this leads to overtreatment or appropriate escalation of care. Nonetheless, MRI provides a more detailed assessment of the local extent of disease than traditional staging by a physical exam, which can inform treatment decisions. MRI, along with PSA and biopsy, is also an important tool in the selection of candidates for active surveillance and for monitoring patients once selected. Finally, MRI may be helpful in assessing for local recurrence in the setting of biochemical failure; however, its diagnostic yield is limited in patients with lower PSAs. Further study is needed in order to solidify how MR findings should be incorporated into risk group stratification and treatment planning, as well as the ideal time to carry out MRI in the setting of biochemical failure.

## 3. The Role of Molecular Imaging in Contemporary Prostate Cancer Management

### 3.1. Overview of Commonly Used Prostate Cancer Molecular Imaging Agents

Several radioligands are available for PET imaging of prostate cancer, leveraging differences in functional pathways and structural cellular components between prostate cancer cells and surrounding tissues. Commonly available radioligands that query metabolic activity include ^11^C-acetate, ^11^C-choline, ^18^F-choline, ^18^F-fluciclovine (Axumin^TM^), and ^18^F sodium fluoride (Na^18^F) [2,30]. Choline is a cell membrane component involved in cell membrane synthesis [31], while acetate is involved in the tricarboxylic acid cycle and lipid synthesis [32]. Choline can be used in PET imaging to detect both osseous and nonosseous lesions with high specificity, although demonstrates sensitivity for nodal metastases [33]. Fluciclovine is a synthetic amino acid radiotracer that has been prospectively evaluated in the Empire I trial and found to have better diagnostic performance than CT and MRI for restaging biochemically recurrent prostate cancer [34]. Na^18^F targets osteoblastic activity where fluoride is being deposited into new bone and is specifically used in imaging osseous metastases [2]. 

Prostate-specific membrane antigen (PSMA) radioligands target a transmembrane zinc metalloenzyme that is upregulated and overexpressed by prostate cancer cells compared with other tissues that contain the protein. These include salivary and lacrimal glands, spleen, liver, small intestine, kidney, pleura, and endometrium [30,31,35,36]. The degree of PSMA expression is reported in a standardized fashion with miPSMA scoring and has been associated with prostate cancer aggressiveness and prognosis [36,37]. There are at least 25 PSMA radiotracers in use, though the most frequently used are ^68^Ga-PSMA-11 and ^18^F-DCFPyL [35]. This approach to molecular imaging provides high specificity compared with MRI, CT, or choline, and a higher sensitivity for nodal disease than other modalities [38,39]. Used in combination, PSMA and metabolic tracers can provide different information about the status of prostate cancer cells, although it should be noted with all PET scanners that metastases smaller than the spatial resolution of the scanners or with activity below the current limits of detection may remain occult [38]. In the future, these limitations in PET imaging may be addressed through improved technology, such as long axial field-of-view (LAFOV) scanners, which can image faster and maintain sensitivity at low radiopharmacceutical activity levels [40], and through improved understanding of target expression optimization [41].

In addition to providing clinical insight for treatment decisions both for upfront treatment as well as at times of recurrence/progression, radioligands useful for diagnostic imaging may be paired with radiopharmaceuticals for theranostic purposes. As an example, ^177^Lu-PSMA or 225Ac-PSMA are promising treatments for PSMA-avid metastatic castrate-resistant prostate cancer [42,43]. ^68^Ga-PSMA-11 imaging is predictive of ^177^Lu-PSMA-617 and ^225^Ac-Lu-PSMA-617 biodistribution and dosimetry, as is ^64^Cu- imaging for ^67^Cu- therapy [44]. Recently, novel ^18^F radiohybrid PSMA ligands (rhPSMA) with two binding sites for radionuclides have been developed [45]. Thus far, ^18^F-rhPSMA-7.3 has been studied for diagnostic purposes, although with future potential to pair with radiohybrid radiopharamaceuticals such as ^177^Lu to bridge ^18^F PET and radioligand therapy [46]. 

### 3.2. Molecular Imaging for Initial Staging of Prostate Cancer

In the upfront staging setting, the sensitivity of PSMA PET and its increasing use in modern practice may allow for careful diagnosis and risk-stratification of patients prior to treatment. Both ^68^Ga-PSMA-11 and ^18^F-DCFPyL are FDA approved for initial staging in the setting of suspected metastatic disease. According to the NCCN guidelines, PSMA PET-CT can be used as an alternative for standard imaging of bone and soft tissue for initial staging [2]. For the detection of metastatic bone disease, standard imaging modalities as well ^11^C-choline or Na^18^F PET are permitted. PSMA imaging is recommended prior to the initiation of androgen deprivation therapy and can be used upfront for unfavorable intermediate and higher risk groups. 

Careful selection of patients prior to treatment initiation as well as the early detection of small regional and/or limited distant metastasis may be possible with molecular imaging. For example, the POP-RT trial required PSMA PET for initial staging due to its higher sensitivity for identifying metastatic disease within and outside the pelvis [47]. This allowed for the inclusion of a selected group of patients with localized high-risk prostate cancer that had not yet progressed detectably beyond the pelvis and who benefitted from whole pelvis radiotherapy with higher rates of biochemical-recurrence-free survival and disease-free survival. 

### 3.3. Assessment of Biochemical Recurrence or High-Risk Postoperative Patients

Following primary treatment, detectable and/or rising PSA following initial treatment is suggestive of biochemical recurrence. Biochemical recurrence historically has been defined either as rising into a detectable range following prostatectomy or after radiotherapy, according to the Phoenix criteria or ASTRO definition. The Phoenix criteria are defined as nadir + 2 ng/mL, while ASTRO specifies biochemical failure as three consecutive PSA rises [48,49]. In this setting, PET imaging may be useful for detecting the foci of recurrent prostate cancer and may influence treatment decisions [50,51,52,53].

Current guidelines, including the AUA and NCCN guidelines, endorse using FDA-approved PSMA tracers for PET-CT as an alternative to conventional CT imaging or bone scans in the setting of biochemical recurrence [2,54]. While choline [55] and fluciclovine [56] are less sensitive than PSMA, especially at low PSA levels, they may also be used to detect low volume recurrent disease and soft tissue and bone. However, due to a lack of evidence, the EAU guidelines currently recommend strongly against changing treatment based on PSMA PET-CT findings [38]. 

There have been several notable clinical trials in this setting using ^18^F-fluciclovine. FALCON was a trial in men with biochemically recurrent prostate cancer who required salvage treatment in the United Kingdom. Initial treatment planning was performed based on standard imaging. Subsequent fluciclovine PET-CT informed revisions of radiation plans in 63% of patients, including the decision to escalate care or de-escalate to watchful waiting [57]. LOCATE was a similar multicenter North American study using fluciclovine PET-CT in patients with biochemical recurrence following initial prostate cancer treatment, reporting that 59% of patients had changes in the initial management plan that were influenced by the scan, with changes that were considered major in 78% of patients [58]. EMPIRE-1 was a phase 2/3 North American study of fluciclovine PET-CT for radiotherapy target volume delineation in patients with biochemically recurrent prostate cancer and negative conventional imaging [34]. This resulted in significantly improved 3-year event-free survival for the fluciclovine group compared with conventional imaging (75.5% versus 63%) and altered treatment-decision making, including targeting distant metastases and offering androgen deprivation therapy in 35%. 

The RAVES trial, comparing adjuvant versus salvage radiotherapy following prostatectomy, allowed PSMA scan utilization to define progression during surveillance [59,60,61]. Exactly how the scan results influenced subsequent treatment was not clearly reported in these studies; however, there is other prospective evidence that ^68^Ga-PSMA-11 has the potential to inform clinical decisions in primary and/or recurrent prostate cancer due to the detection of previously unsuspected locoregional or distant disease, especially in the setting of biochemical recurrence [62]. 

### 3.4. Interpretation of Post-Treatment Molecular Imaging

While treatment response measured by choline occurs in a fairly predictable, well-documented fashion [63], interpretation and optimal timing of post-treatment PSMA imaging are not currently well-defined [64]. Following metastasis-directed radiotherapy, a partial or complete PSMA-PET response may be seen 3–6 months after treatment in the majority of patients with associated improved metastasis-free survival [65]. Consensus guidelines regarding the utilization of PET for assessing treatment response are lacking.

Progression and/or recurrence is typically defined as the appearance of new lesions or growth of existing lesions. With PSMA imaging, it has been proposed that at least two new distant lesions are required to define disease progression to reduce confounding by false-positive results, one new PSMA positive lesions with suggestive laboratory data, and/or an increase by 30% in the size/uptake with suggestive laboratory data [64]. 

### 3.5. Unexpected Findings

There have been reports in a small subset of prostate cancer patients of discordant surveillance imaging findings. For example, malignant findings with choline and/or PSMA PET avidity may be seen at low PSA levels. Garg et al. published a series of patients with a history of treated BCR and PSA less than or equal to 0.1 ng/mL undergoing surveillance choline imaging and found that 13/107 (12.1%) demonstrated positive surveillance imaging with distant metastases and local recurrences that were confirmed with gold standard radiologic/pathologic tests [66]. There were no false-positive or negative findings reported. These findings suggested that for late-stage disease subject to multiple clinical courses, choline PET may allow for the detection of relapse at undetectable PSA levels. It is also known that neuroendocrine differentiation of prostate cancer can render it non PSMA-avid and result in false negative scan results. This process tends to occur in patients who have received prolonged ADT and/or other systemic agents and is often associated with aggressive behavior, including treatment resistance and rapid progression of disease [36]. The choline PET findings in the study by Garg et al. were hypothesized to be related to small cell or neuroendocrine differentiation, active ADT, and/or selective non-PSA-producing disease following prolonged chemohormonal therapy [66,67]. Close attention to changes in CT or MRI appearance in the absence of PSMA or choline signals as well as FDG PET may be critical in the identification of non-radiotracer-avid lesions in these situations and it remains unclear what role, if any, PSMA radioligand monotherapy may play in the treatment of patients with non-PSMA-expressing lesions that may be underdosed or untreated [42,68].

There have also been increasing reports of false-positive and/or negative results beyond the normal biodistribution with PSMA PET-CT due to non-prostatic malignancies, either in benign or malignant settings [36]. This can be particularly problematic in the oligometastatic or oligo recurrent setting, where a solitary false-positive result can potentially lead to misunderstanding the extent of the disease. Commonly, this can occur due to the physiologic radiotracer uptake in nervous tissue of the sympathetic chain, as well as cervical celiac and sacral ganglia, which in the pelvis may mimic nodal metastases and confound treatment decision making [36]. PSMA can also be expressed in the endothelium and/or other tissues in benign proliferative processes, such as keloids, granulation tissue in heart valves, or gynecomastia [36,69]. Benign pathologic processes, including granulomatous disease, can present with avid pulmonary lesions and/or mediastinal adenopathy; benign bone pathology such as Paget’s disease, healing fractures, fibrous dysplasia, various soft tissue lesions such as hemangiomas, desmoid tumors, dermatofibromas and cerebral infarction can all display false-positive PSMA uptake [36]. 

It may be possible to identify benign, non-prostate cancer PSMA-avid lesions without biopsy. For example, in a study of patients with newly diagnosed prostate cancer and solitary PSMA-avid rib lesions on initial staging imaging, the primary prostate was treated either surgically or with radiotherapy, and serial surveillance imaging was carried out for rib lesions, with the following criteria for benign lesions: stable size or undetectable/at nadir PSA. Ultimately, 98.4% of men in this study met these criteria for having benign lesions, and there was only a single false-negative, malignant lesion [70]. 

In non-prostate neoplasms, PSMA uptake can also be associated with benign or malignant tumor neo-vasculature. This can be seen in neurogenic neoplasms such as meningioma or malignant pathologies such as glioblastoma, follicular lymphoma, multiple myeloma, pancreatic neuroendocrine tumors, and certain carcinomas arising from the head and neck, lung, breast, genitourinary, or gastrointestinal tracts [36]. 

Even prostate cancer cells themselves are not always PSMA avid, and multiple prostate cancer lesions may be identifiable as non-overlapping lesions with varying avidity for different radiotracers. For example, PSMA PET outperforms ^11^C-acetate diagnostically; however, while most lesions are both PSMA and ^11^C-acetate avid, 38% of PSMA avid metastases lack ^11^C-acetate uptake and are metabolically inactive. It has also been observed that 15% of metabolically active ^11^C-acetate positive metastasis do not express PSMA [30]. Thus, unexpected findings on molecular imaging should be considered in the context of the patient’s cancer history as well as other co-morbid conditions, with consideration for biopsy, surveillance, and/or repeat imaging with alternative radiotracers. 

### 3.6. Ongoing Considerations for Prostate Molecular Imaging

In an era of increasingly sensitive molecular imaging, the foci of recurrent disease can be detected much earlier than was historically possible. This raises several ongoing concerns. The first is for “stage migration” or “stage creep”, in which patients historically classified into earlier stage groups might be upstaged [2]. Ultimately, this can contribute to changes in survival outcomes in both stage groups in a process termed the “Will Rogers Phenomenon” [71,72]. This has already been observed in prostate cancer in the 1990s; gradual change in the application of the Gleason score over time resulted in a decline in the reported incidence of low-grade prostate cancer and was found to be associated with a 28% lower mortality rate according to the Gleason score compared with historical data [73]. With increasing identification of oligometastatic cancer and recognition as a potentially curable, intermediate entity between patients with widely metastatic prostate cancer with a historically poor prognosis and localized prostate cancer, there is the possibility a similar phenomenon is again occurring [74]. 

Second, while it is true for PSMA, Axumin, and F18 tracers that there is an increasing detection rate at higher PSA levels, it is possible for disease detection prior to meeting criteria for biochemical recurrence [33,39,58]. For example, in a cohort of patients with recurrent prostate cancer diagnosed on choline PET-CT, 71% of those who had imaging prior to meeting the Phoenix criteria had identifiable foci of disease [75]. Compared with choline, PSMA has a higher sensitivity for detecting prostate cancer and is less likely to underestimate tumor burden, even at very low PSA levels [2,76,77]. Additionally, in direct prospective comparison with fluciclovine, PSMA was associated with better detection rates of prostate cancer overall, although fluciclovine outperformed it in the prostate bed where urinary accumulation of the PSMA-tracer can conceal local recurrence [56,78]. Thus, with the use of PSMA and/or fluciclovine scans, there is a good likelihood of detecting an even greater number of recurrences. A modern definition of biochemical recurrence should be considered to account for the current molecular imaging capabilities.

Third, there is sparse evidence on how using PET scans may impact patient outcomes. Thus far, the EMPIRE-I trial utilizing fluciclovine is the only study in this setting with oncologic outcomes as the endpoint [34]. The ongoing EMPIRE-2 study (clinicaltrials.gov registration: NCT03762759, accessed on 31 October 2023) seeks to perform a randomized comparison of PSMA with fluciclovine with oncologic endpoints, and an ongoing FDA initiative to study “the impact of novel PET imaging tracers on real-world outcomes for patients with prostate cancer” will hopefully provide further insight [34,79]. 

Ultimately, how best to use the results of molecular imaging in treatment decision making upfront as well as in salvage treatment, respond to concerns for stage migration, re-define biochemical recurrence in the modern era, and how to best use molecular imaging for surveillance, particularly in late-stage metastatic castrate-resistant disease, should be the subject of future investigation. It has been suggested that for future clinical studies, radiographic-progression-free survival should be used as the surrogate oncologic endpoint for overall survival [80,81]. Integration of molecular imaging into this endpoint as well as using molecular imaging to define patient inclusion for contemporary trials may provide added clarity over time. Radiomics in molecular imaging is emerging for a variety of purposes, including image reconstruction, tumor and/or metastasis segmentation, as well as the characterization of lesions and to identify responses to treatment. It may be possible to utilize data from future trials to develop clinically useful applications of radiomics and artificial intelligence tools [82].

## 4. Conclusions

MRI and prostate cancer-specific PET represent two widely applicable, rapidly developing technologies that are becoming increasingly important to prostate cancer diagnosis and management. While the adoption of these techniques will help us make the most informed decisions with our patients, it is important to recognize that the clinical benefits and cost-effectiveness of their use are still being evaluated and debated. With continued prospective evaluation, advanced imaging for prostate cancer will continue to improve the personalization and efficiency of care.

## Figures and Tables

**Table 1:** Uses of MRI and PET in various aspects of prostate cancer care.

	Initial Diagnosis	Staging and Risk Stratification	Treatment Selection	Active Surveillance	Recurrence Detection	Treatment Response Assessment
MRI	Fusion biopsy improves clinically significant cancer detection when used as an adjunct to standard biopsy.	Assesses local extent of disease and nodal involvement. If incorporated into staging, it often upstages.	Upstaging and treatment escalation based on MR findings are controversial but increasingly common.	Fundamental in assessment of PSA or physical exam change and to guide subsequent repeat biopsy.	Assesses local recurrence. Recurrence detection improved at higher PSAs. Ideal timing of obtaining MRI is unclear.	Currently difficult to interpret in isolation but continues to evolve.
Prostate-specific PET	Limited use/not applicable.	Primarily assesses nodal and distant metastatic disease. May be used as alternative for standard imaging of bone and soft tissue in initial staging. May also inform local disease distribution.	Presence or absence of PET-detected metastatic disease influences local vs. systemic treatment approach.	Limited use/not applicable.	Assesses local and systemic recurrence. PSMA is more sensitive than choline and fluciclovine at low PSA levels; however, there is no/limited evidence supporting treatment changes based on PSMA findings.	Choline PET is the most consistently interpretable while response assessment by PSMA remains incompletely understood.

## Data Availability

Not applicable.

## Abbreviations

AJCC	American Joint Committee on Cancer
AS	Active Surveillance
ASTRO	American Society for Radiation Oncology
AUA	American Urological Association
AUC	Area Under the Curve
BCR	Biochemical Recurrence
BRFS	Biochemical Recurrence-Free Survival
CT	Computed Tomography
DRE	Digital Rectal Exam
EAU	European Association of Urology
FDA	Food and Drug Administration
MR	Magnetic Resonance
MRI	Magnetic Resonance Imaging
NCCN	National Comprehensive Cancer Network
NCI	National Cancer Institute
PET	Positron Emission Tomography
PIRADS	Prostate Imaging Reporting & Data System
PSA	Prostate-Specific Antigen
PSMA	Prostate-Specific Membrane Antigen
T2W	T2-Weighted
TRUS	Trans-Rectal Ultrasound
UK	United Kingdom
US	United States

## References

[1] Siegel R.L., Miller K.D., Wagle N.S., Jemal A. (2023). Cancer statistics, 2023. CA Cancer J. Clin..

[2] Schaeffer E.M., Srinivas S., Adra N., An Y., Barocas D., Bitting R., Bryce A., Chapin B., Cheng H.H., D’Amico A.V. (2023). Prostate Cancer, Version 4.2023. J. Natl. Compr. Cancer Netw..

[3] Eastham J.A., Auffenberg G.B., Barocas D.A., Chou R., Crispino T., Davis J.W., Eggener S., Horwitz E.M., Kane C.J., Kirkby E. (2022). Clinically Localized Prostate Cancer: AUA/ASTRO Guideline, Part I: Introduction, Risk Assessment, Staging, and Risk-Based Management. J. Urol..

[4] Mottet N., van den Bergh R.C.N., Briers E., Van den Broeck T., Cumberbatch M.G., De Santis M., Fanti S., Fossati N., Gandaglia G., Gillessen S. (2021). EAU-EANM-ESTRO-ESUR-SIOG Guidelines on Prostate Cancer-2020 Update. Part 1: Screening, Diagnosis, and Local Treatment with Curative Intent. Eur. Urol..

[5] Dasgupta P., Davis J., Hughes S. (2019). NICE guidelines on prostate cancer 2019. BJU Int..

[6] Kasivisvanathan V., Rannikko A.S., Borghi M., Panebianco V., Mynderse L.A., Vaarala M.H., Briganti A., Budaus L., Hellawell G., Hindley R.G. (2018). MRI-Targeted or Standard Biopsy for Prostate-Cancer Diagnosis. N. Engl. J. Med..

[7] Siddiqui M.M., Rais-Bahrami S., Turkbey B., George A.K., Rothwax J., Shakir N., Okoro C., Raskolnikov D., Parnes H.L., Linehan W.M. (2015). Comparison of MR/ultrasound fusion-guided biopsy with ultrasound-guided biopsy for the diagnosis of prostate cancer. JAMA.

[8] Rouviere O., Puech P., Renard-Penna R., Claudon M., Roy C., Mege-Lechevallier F., Decaussin-Petrucci M., Dubreuil-Chambardel M., Magaud L., Remontet L. (2019). Use of prostate systematic and targeted biopsy on the basis of multiparametric MRI in biopsy-naive patients (MRI-FIRST): A prospective, multicentre, paired diagnostic study. Lancet Oncol..

[9] Amin M.B., Amin M.B., Edge S.B., Greene F.L., American Joint Committee on Cancer, American Cancer Society (2017). AJCC Cancer Staging Manual.

[10] Parker C.C., James N.D., Brawley C.D., Clarke N.W., Hoyle A.P., Ali A., Ritchie A.W.S., Attard G., Chowdhury S., Cross W. (2018). Radiotherapy to the primary tumour for newly diagnosed, metastatic prostate cancer (STAMPEDE): A randomised controlled phase 3 trial. Lancet.

[11] Soeterik T.F.W., van Melick H.H.E., Dijksman L.M., Biesma D.H., Witjes J.A., van Basten J.A. (2021). Multiparametric Magnetic Resonance Imaging Should Be Preferred Over Digital Rectal Examination for Prostate Cancer Local Staging and Disease Risk Classification. Urology.

[12] Draulans C., Everaerts W., Isebaert S., Gevaert T., Oyen R., Joniau S., Lerut E., De Wever L., Weynand B., Vanhoutte E. (2019). Impact of Magnetic Resonance Imaging on Prostate Cancer Staging and European Association of Urology Risk Classification. Urology.

[13] Smith D.S., Catalona W.J. (1995). Interexaminer variability of digital rectal examination in detecting prostate cancer. Urology.

[14] Wen J., Ji Y., Han J., Shen X., Qiu Y. (2022). Inter-reader agreement of the prostate imaging reporting and data system version v2.1 for detection of prostate cancer: A systematic review and meta-analysis. Front. Oncol..

[15] Ball M.W., Partin A.W., Epstein J.I. (2015). Extent of extraprostatic extension independently influences biochemical recurrence-free survival: Evidence for further pT3 subclassification. Urology.

[16] Sung M.T., Lin H., Koch M.O., Davidson D.D., Cheng L. (2007). Radial distance of extraprostatic extension measured by ocular micrometer is an independent predictor of prostate-specific antigen recurrence: A new proposal for the substaging of pT3a prostate cancer. Am. J. Surg. Pathol..

[17] Flegar L., Zacharis A., Aksoy C., Heers H., Derigs M., Eisenmenger N., Borkowetz A., Groeben C., Huber J. (2022). Alternative- and focal therapy trends for prostate cancer: A total population analysis of in-patient treatments in Germany from 2006 to 2019. World J. Urol..

[18] Stamatakis L., Siddiqui M.M., Nix J.W., Logan J., Rais-Bahrami S., Walton-Diaz A., Hoang A.N., Vourganti S., Truong H., Shuch B. (2013). Accuracy of multiparametric magnetic resonance imaging in confirming eligibility for active surveillance for men with prostate cancer. Cancer.

[19] Ouzzane A., Renard-Penna R., Marliere F., Mozer P., Olivier J., Barkatz J., Puech P., Villers A. (2015). Magnetic Resonance Imaging Targeted Biopsy Improves Selection of Patients Considered for Active Surveillance for Clinically Low Risk Prostate Cancer Based on Systematic Biopsies. J. Urol..

[20] Eineluoto J.T., Jarvinen P., Kenttamies A., Kilpelainen T.P., Vasarainen H., Sandeman K., Erickson A., Mirtti T., Rannikko A. (2017). Repeat multiparametric MRI in prostate cancer patients on active surveillance. PLoS ONE.

[21] Walton Diaz A., Shakir N.A., George A.K., Rais-Bahrami S., Turkbey B., Rothwax J.T., Stamatakis L., Hong C.W., Siddiqui M.M., Okoro C. (2015). Use of serial multiparametric magnetic resonance imaging in the management of patients with prostate cancer on active surveillance. Urol. Oncol..

[22] Chesnut G.T., Vertosick E.A., Benfante N., Sjoberg D.D., Fainberg J., Lee T., Eastham J., Laudone V., Scardino P., Touijer K. (2020). Role of Changes in Magnetic Resonance Imaging or Clinical Stage in Evaluation of Disease Progression for Men with Prostate Cancer on Active Surveillance. Eur. Urol..

[23] Midiri F., Vernuccio F., Purpura P., Alongi P., Bartolotta T.V. (2021). Multiparametric MRI and Radiomics in Prostate Cancer: A Review of the Current Literature. Diagnostics.

[24] Recabal P., Assel M., Sjoberg D.D., Lee D., Laudone V.P., Touijer K., Eastham J.A., Vargas H.A., Coleman J., Ehdaie B. (2016). The Efficacy of Multiparametric Magnetic Resonance Imaging and Magnetic Resonance Imaging Targeted Biopsy in Risk Classification for Patients with Prostate Cancer on Active Surveillance. J. Urol..

[25] Casciani E., Polettini E., Carmenini E., Floriani I., Masselli G., Bertini L., Gualdi G.F. (2008). Endorectal and dynamic contrast-enhanced MRI for detection of local recurrence after radical prostatectomy. AJR Am. J. Roentgenol..

[26] Kim M., Hwang S.I., Ahn H., Lee H.J., Byun S.S., Hong S.K., Lee S. (2022). Diagnostic yield of multiparametric MRI for local recurrence at biochemical recurrence after radical prostatectomy. Prostate Int..

[27] Figler B.D., Reuther A.M., Dhar N., Levin H., Magi-Galluzzi C., Zhou M., Klein E.A. (2007). Preoperative PSA is still predictive of cancer volume and grade in late PSA era. Urology.

[28] Westphalen A.C., Kurhanewicz J., Cunha R.M., Hsu I.C., Kornak J., Zhao S., Coakley F.V. (2009). T2-Weighted endorectal magnetic resonance imaging of prostate cancer after external beam radiation therapy. Int. Braz. J. Urol..

[29] Sala E., Eberhardt S.C., Akin O., Moskowitz C.S., Onyebuchi C.N., Kuroiwa K., Ishill N., Zelefsky M.J., Eastham J.A., Hricak H. (2006). Endorectal MR imaging before salvage prostatectomy: Tumor localization and staging. Radiology.

[30] Regula N., Kostaras V., Johansson S., Trampal C., Lindstrom E., Lubberink M., Velikyan I., Sorensen J. (2020). Comparison of (68)Ga-PSMA-11 PET/CT with (11)C-acetate PET/CT in re-staging of prostate cancer relapse. Sci. Rep..

[31] Evans J.D., Jethwa K.R., Ost P., Williams S., Kwon E.D., Lowe V.J., Davis B.J. (2018). Prostate cancer-specific PET radiotracers: A review on the clinical utility in recurrent disease. Pract. Radiat. Oncol..

[32] Czernin J., Benz M.R., Allen-Auerbach M.S. (2009). PET Imaging of Prostate Cancer Using C-Acetate. PET Clin..

[33] Michaud L., Touijer K.A., Mauguen A., Zelefsky M.J., Morris M.J., Lyashschenko S.K., Durack J.C., Humm J.L., Weber W.A., Schoder H. (2020). (11)C-Choline PET/CT in Recurrent Prostate Cancer: Retrospective Analysis in a Large U.S. Patient Series. J. Nucl. Med..

[34] Jani A.B., Schreibmann E., Goyal S., Halkar R., Hershatter B., Rossi P.J., Shelton J.W., Patel P.R., Xu K.M., Goodman M. (2021). (18)F-fluciclovine-PET/CT imaging versus conventional imaging alone to guide postprostatectomy salvage radiotherapy for prostate cancer (EMPIRE-1): A single centre, open-label, phase 2/3 randomised controlled trial. Lancet.

[35] Zippel C., Ronski S.C., Bohnet-Joschko S., Giesel F.L., Kopka K. (2020). Current Status of PSMA-Radiotracers for Prostate Cancer: Data Analysis of Prospective Trials Listed on ClinicalTrials.gov. Pharmaceuticals.

[36] Sheikhbahaei S., Afshar-Oromieh A., Eiber M., Solnes L.B., Javadi M.S., Ross A.E., Pienta K.J., Allaf M.E., Haberkorn U., Pomper M.G. (2017). Pearls and pitfalls in clinical interpretation of prostate-specific membrane antigen (PSMA)-targeted PET imaging. Eur. J. Nucl. Med. Mol. Imaging.

[37] Eiber M., Herrmann K., Calais J., Hadaschik B., Giesel F.L., Hartenbach M., Hope T., Reiter R., Maurer T., Weber W.A. (2018). Prostate Cancer Molecular Imaging Standardized Evaluation (PROMISE): Proposed miTNM Classification for the Interpretation of PSMA-Ligand PET/CT. J. Nucl. Med..

[38] Cornford P., Briers E., Eberli D., Oldenburg J., Rouviere O., Tilki D., Oort I.M.V., Farolfi A., Lardas M., Broeck T.V.d. EAU Prostate Cancer Guidelines. http://uroweb.org/guidelines/compilations-of-all-guidelines/.

[39] Perera M., Papa N., Christidis D., Wetherell D., Hofman M.S., Murphy D.G., Bolton D., Lawrentschuk N. (2016). Sensitivity, Specificity, and Predictors of Positive (68)Ga-Prostate-specific Membrane Antigen Positron Emission Tomography in Advanced Prostate Cancer: A Systematic Review and Meta-analysis. Eur. Urol..

[40] Filippi L., Dimitrakopoulou-Strauss A., Evangelista L., Schillaci O. (2022). Long axial field-of-view PET/CT devices: Are we ready for the technological revolution?. Expert Rev. Med. Devices.

[41] Hope T.A., Truillet C., Ehman E.C., Afshar-Oromieh A., Aggarwal R., Ryan C.J., Carroll P.R., Small E.J., Evans M.J. (2017). 68Ga-PSMA-11 PET Imaging of Response to Androgen Receptor Inhibition: First Human Experience. J. Nucl. Med..

[42] Sartor O., de Bono J., Chi K.N., Fizazi K., Herrmann K., Rahbar K., Tagawa S.T., Nordquist L.T., Vaishampayan N., El-Haddad G. (2021). Lutetium-177-PSMA-617 for Metastatic Castration-Resistant Prostate Cancer. N. Engl. J. Med..

[43] Sathekge M., Bruchertseifer F., Vorster M., Lawal I.O., Knoesen O., Mahapane J., Davis C., Mdlophane A., Maes A., Mokoala K. (2022). mCRPC Patients Receiving (225)Ac-PSMA-617 Therapy in the Post-Androgen Deprivation Therapy Setting: Response to Treatment and Survival Analysis. J. Nucl. Med..

[44] McInnes L.E., Cullinane C., Roselt P.D., Jackson S., Blyth B.J., van Dam E.M., Zia N.A., Harris M.J., Hicks R.J., Donnelly P.S. (2021). Therapeutic Efficacy of a Bivalent Inhibitor of Prostate-Specific Membrane Antigen Labeled with (67)Cu. J. Nucl. Med..

[45] Wurzer A., Di Carlo D., Schmidt A., Beck R., Eiber M., Schwaiger M., Wester H.J. (2020). Radiohybrid Ligands: A Novel Tracer Concept Exemplified by (18)F- or (68)Ga-Labeled rhPSMA Inhibitors. J. Nucl. Med..

[46] Surasi D.S., Eiber M., Maurer T., Preston M.A., Helfand B.T., Josephson D., Tewari A.K., Somford D.M., Rais-Bahrami S., Koontz B.F. (2023). Diagnostic Performance and Safety of Positron Emission Tomography with (18)F-rhPSMA-7.3 in Patients with Newly Diagnosed Unfavourable Intermediate- to Very-high-risk Prostate Cancer: Results from a Phase 3, Prospective, Multicentre Study (LIGHTHOUSE). Eur. Urol..

[47] Murthy V., Maitre P., Kannan S., Panigrahi G., Krishnatry R., Bakshi G., Prakash G., Pal M., Menon S., Phurailatpam R. (2021). Prostate-only versus whole-pelvic radiation therapy in high-risk and very high-risk prostate cancer (POP-RT): Outcomes from phase III randomized controlled trial. J. Clin. Oncol..

[48] Cookson M.S., Aus G., Burnett A.L., Canby-Hagino E.D., D’Amico A.V., Dmochowski R.R., Eton D.T., Forman J.D., Goldenberg S.L., Hernandez J. (2007). Variation in the definition of biochemical recurrence in patients treated for localized prostate cancer: The American Urological Association Prostate Guidelines for Localized Prostate Cancer Update Panel report and recommendations for a standard in the reporting of surgical outcomes. J. Urol..

[49] Abramowitz M.C., Li T., Buyyounouski M.K., Ross E., Uzzo R.G., Pollack A., Horwitz E.M. (2008). The Phoenix definition of biochemical failure predicts for overall survival in patients with prostate cancer. Cancer.

[50] Supiot S., Vaugier L., Pasquier D., Buthaud X., Magne N., Peiffert D., Sargos P., Crehange G., Pommier P., Loos G. (2021). OLIGOPELVIS GETUG P07, a Multicenter Phase II Trial of Combined High-dose Salvage Radiotherapy and Hormone Therapy in Oligorecurrent Pelvic Node Relapses in Prostate Cancer. Eur. Urol..

[51] De Bruycker A., Spiessens A., Dirix P., Koutsouvelis N., Semac I., Liefhooghe N., Gomez-Iturriaga A., Everaerts W., Otte F., Papachristofilou A. (2020). PEACE V—Salvage Treatment of OligoRecurrent nodal prostate cancer Metastases (STORM): A study protocol for a randomized controlled phase II trial. BMC Cancer.

[52] Decaestecker K., De Meerleer G., Ameye F., Fonteyne V., Lambert B., Joniau S., Delrue L., Billiet I., Duthoy W., Junius S. (2014). Surveillance or metastasis-directed Therapy for OligoMetastatic Prostate cancer recurrence (STOMP): Study protocol for a randomized phase II trial. BMC Cancer.

[53] Kneebone A., Hruby G., Ainsworth H., Byrne K., Brown C., Guo L., Guminski A., Eade T. (2018). Stereotactic Body Radiotherapy for Oligometastatic Prostate Cancer Detected via Prostate-specific Membrane Antigen Positron Emission Tomography. Eur. Urol. Oncol..

[54] Lowrance W., Breau R., Chou R., Chapin B.F., Crispino T., Dreicer R., Jarrard D.F., Kibel A.S., Morgan T.M., Morgans A.K. (2023). Updates to advanced prostate cancer: AUA/SUO guidelines. J. Urol..

[55] Treglia G., Pereira Mestre R., Ferrari M., Bosetti D.G., Pascale M., Oikonomou E., De Dosso S., Jermini F., Prior J.O., Roggero E. (2019). Radiolabelled choline versus PSMA PET/CT in prostate cancer restaging: A meta-analysis. Am. J. Nucl. Med. Mol. Imaging.

[56] Calais J., Ceci F., Eiber M., Hope T.A., Hofman M.S., Rischpler C., Bach-Gansmo T., Nanni C., Savir-Baruch B., Elashoff D. (2019). (18)F-fluciclovine PET-CT and (68)Ga-PSMA-11 PET-CT in patients with early biochemical recurrence after prostatectomy: A prospective, single-centre, single-arm, comparative imaging trial. Lancet Oncol..

[57] Scarsbrook A.F., Bottomley D., Teoh E.J., Bradley K.M., Payne H., Afaq A., Bomanji J., As N.v., Chua S., Hoskin P. (2020). Effect of 18F-fluciclovine Positron Emission Tomography on the Management of Patients With Recurrence of Prostate Cancer: Results From the FALCON Trial. Int. J. Radiat. Biol. Phys..

[58] Andriole G.L., Kostakoglu L., Chau A., Duan F., Mahmood U., Mankoff D.A., Schuster D.M., Siegel B.A., Group L.S. (2019). The Impact of Positron Emission Tomography with 18F-Fluciclovine on the Treatment of Biochemical Recurrence of Prostate Cancer: Results from the LOCATE Trial. J. Urol..

[59] Kneebone A., Fraser-Browne C., Duchesne G.M., Fisher R., Frydenberg M., Herschtal A., Williams S.G., Brown C., Delprado W., Haworth A. (2020). Adjuvant radiotherapy versus early salvage radiotherapy following radical prostatectomy (TROG 08.03/ANZUP RAVES): A randomised, controlled, phase 3, non-inferiority trial. Lancet Oncol..

[60] Sargos P., Chabaud S., Latorzeff I., Magne N., Benyoucef A., Supiot S., Pasquier D., Abdiche M.S., Gilliot O., Graff-Cailleaud P. (2020). Adjuvant radiotherapy versus early salvage radiotherapy plus short-term androgen deprivation therapy in men with localised prostate cancer after radical prostatectomy (GETUG-AFU 17): A randomised, phase 3 trial. Lancet Oncol..

[61] Parker C.C., Clarke N.W., Cook A.D., Kynaston H.G., Petersen P.M., Catton C., Cross W., Logue J., Parulekar W., Payne H. (2020). Timing of radiotherapy after radical prostatectomy (RADICALS-RT): A randomised, controlled phase 3 trial. Lancet.

[62] Roach P.J., Francis R., Emmett L., Hsiao E., Kneebone A., Hruby G., Eade T., Nguyen Q.A., Thompson B.D., Cusick T. (2018). The Impact of (68)Ga-PSMA PET/CT on Management Intent in Prostate Cancer: Results of an Australian Prospective Multicenter Study. J. Nucl. Med..

[63] Alongi P., Laudicella R., Lanzafame H., Farolfi A., Mapelli P., Picchio M., Burger I.A., Iagaru A., Minutoli F., Evangelista L. (2022). PSMA and Choline PET for the Assessment of Response to Therapy and Survival Outcomes in Prostate Cancer Patients: A Systematic Review from the Literature. Cancers.

[64] Fanti S., Hadaschik B., Herrmann K. (2020). Proposal for Systemic-Therapy Response-Assessment Criteria at the Time of PSMA PET/CT Imaging: The PSMA PET Progression Criteria. J. Nucl. Med..

[65] Sutera P., Deek M.P., Guler O.C., Hurmuz P., Reyhan M., Rowe S., Hrinivich W.T., Ren L., Song D., Kiss A.P. (2022). Prostate-specific membrane antigen PET response associates with metastasis-free survival following stereotactic ablative radiation therapy in oligometastatic castration-sensitive prostate cancer. Int. J. Radiat. Oncol. Biol. Phys..

[66] Garg I., Nathan M.A., Packard A.T., Kwon E.D., Larson N.B., Lowe V., Davis B.J., Haloi R., Mahon M.L., Goenka A.H. (2021). (11)C-choline positron emission tomography/computed tomography for detection of disease relapse in patients with history of biochemically recurrent prostate cancer and prostate-specific antigen </=0.1 ng/ml. J. Cancer Res. Ther..

[67] Bryce A.H., Alumkal J.J., Armstrong A., Higano C.S., Iversen P., Sternberg C.N., Rathkopf D., Loriot Y., de Bono J., Tombal B. (2017). Radiographic progression with nonrising PSA in metastatic castration-resistant prostate cancer: Post hoc analysis of PREVAIL. Prostate Cancer Prostatic Dis..

[68] Michalski K., Ruf J., Goetz C., Seitz A.K., Buck A.K., Lapa C., Hartrampf P.E. (2021). Prognostic implications of dual tracer PET/CT: PSMA ligand and [(18)F]FDG PET/CT in patients undergoing [(177)Lu]PSMA radioligand therapy. Eur. J. Nucl. Med. Mol. Imaging.

[69] Sasikumar A., Joy A., Nair B.P., Pillai M.R.A., Madhavan J. (2017). False Positive Uptake in Bilateral Gynecomastia on 68Ga-PSMA PET/CT Scan. Clin. Nucl. Med..

[70] Chen M.Y., Franklin A., Yaxley J., Gianduzzo T., McBean R., Wong D., Tatkovic A., McEwan L., Walters J., Kua B. (2020). Solitary rib lesions showing prostate-specific membrane antigen (PSMA) uptake in pre-treatment staging (68) Ga-PSMA-11 positron emission tomography scans for men with prostate cancer: Benign or malignant?. BJU Int..

[71] Feinstein A.R., Sosin D.M., Wells C.K. (1985). The Will Rogers Phenomenon. N. Engl. J. Med..

[72] Sormani M.P. (2009). The Will Rogers Phenomenon: The effect of different diagnostic criteria. J. Neurol. Sci..

[73] Albertsen P.C., Hanley J.A., Barrows G.H., Penson D.F., Kowalczyk P.D., Sanders M.M., Fine J. (2005). Prostate cancer and the Will Rogers phenomenon. J. Natl. Cancer Inst..

[74] Connor M.J., Winkler M., Ahmed H.U. (2020). Survival in Oligometastatic Prostate Cancer-A New Dawn or the Will Rogers Phenomenon?. JAMA Oncol..

[75] Parker W.P., Davis B.J., Park S.S., Olivier K.R., Choo R., Nathan M.A., Lowe V.J., Welch T.J., Evans J.D., Harmsen W.S. (2017). Identification of Site-specific Recurrence Following Primary Radiation Therapy for Prostate Cancer Using C-11 Choline Positron Emission Tomography/Computed Tomography: A Nomogram for Predicting Extrapelvic Disease. Eur. Urol..

[76] Dietlein F., Kobe C., Neubauer S., Schmidt M., Stockter S., Fischer T., Schomacker K., Heidenreich A., Zlatopolskiy B.D., Neumaier B. (2017). PSA-Stratified Performance of (18)F- and (68)Ga-PSMA PET in Patients with Biochemical Recurrence of Prostate Cancer. J. Nucl. Med..

[77] Fossati N., Scarcella S., Gandaglia G., Suardi N., Robesti D., Boeri L., Karnes R.J., Heidenreich A., Pfister D., Kretschmer A. (2020). Underestimation of Positron Emission Tomography/Computerized Tomography in Assessing Tumor Burden in Prostate Cancer Nodal Recurrence: Head-to-Head Comparison of (68)Ga-PSMA and (11)C-Choline in a Large, Multi-Institutional Series of Extended Salvage Lymph Node Dissections. J. Urol..

[78] Pernthaler B., Kulnik R., Gstettner C., Salamon S., Aigner R.M., Kvaternik H. (2019). A Prospective Head-to-Head Comparison of 18F-Fluciclovine With 68Ga-PSMA-11 in Biochemical Recurrence of Prostate Cancer in PET/CT. Clin. Nucl. Med..

[79] Leapman M., Ross J., Jeffery M., Gross C., Saperstein L., Karnes R.J., Kunst N., Ma X., Wang S.Y., Long J. The Impact of Novel Positron Emission Tomography (PET) Imaging Tracers on Real-World Outcomes for Patients with Prostate Cancer. https://www.fda.gov/science-research/advancing-regulatory-science/impact-novel-positron-emission-tomography-pet-imaging-tracers-real-world-outcomes-patients-prostate.

[80] Xie W., Regan M.M., Buyse M., Halabi S., Kantoff P.W., Sartor O., Soule H., Clarke N.W., Collette L., Dignam J.J. (2017). Metastasis-Free Survival is a Strong Surrogate of Overall Survival in Localized Prostate CAncer. J. Clin. Oncol..

[81] Halabi S., Roy A., Rydzewska L., Godolphin P., Parmar M.K., Hussain M.H., Tangen C., Thompson I., Xie W., Carducci M.A. (2022). Assessing intermediate clinical endpoints (ICE) as potential surrogates for overall survival (OS) in men with metastatic hormone-sensitive prostate cancer (mHSPC). J. Clin. Oncol..

[82] Liberini V., Laudicella R., Balma M., Nicolotti D.G., Buschiazzo A., Grimaldi S., Lorenzon L., Bianchi A., Peano S., Bartolotta T.V. (2022). Radiomics and artificial intelligence in prostate cancer: New tools for molecular hybrid imaging and theragnostics. Eur. Radiol. Exp..

